# Mass testing and treatment for malaria in low transmission areas in Amhara Region, Ethiopia

**DOI:** 10.1186/s12936-016-1333-3

**Published:** 2016-06-02

**Authors:** Callie A. Scott, Asnakew K. Yeshiwondim, Belendia Serda, Caterina Guinovart, Berhane H. Tesfay, Adem Agmas, Melkamu T. Zeleke, Girma S. Guesses, Asmamaw L. Ayenew, Worku M. Workie, Richard W. Steketee, Duncan Earle, Belay Bezabih, Asefaw Getachew

**Affiliations:** PATH Malaria Control and Elimination Partnership in Africa (MACEPA), 2201 Westlake Avenue, Suite 200, Seattle, WA 98121 USA; PATH Malaria Control and Elimination Partnership in Africa (MACEPA), Getu Commercial Center, Rear Side, 1st–4th Floors, PO Box 493, 1110 Addis Ababa, Ethiopia; PATH Malaria Control and Elimination Partnership in Africa (MACEPA), Stand #16806, Trinity Park, Alick Nkhata Road, Mass Media Area, Lusaka, 10101 Zambia; Amhara National Regional State Health Bureau, P.O. Box 495, Bahir Dar, Ethiopia

**Keywords:** Malaria, Mass test and treat, *Plasmodium falciparum*, *Plasmodium vivax*, Ethiopia

## Abstract

**Background:**

In areas with ongoing malaria transmission, strategies to clear parasites from populations can reduce infection and transmission. The objective of this paper was to describe a malaria mass testing and treatment (MTAT) intervention implemented in six *kebeles* (villages) in Amhara Region, Ethiopia, at the beginning of the 2014 transmission season.

**Methods:**

Intervention *kebeles* were selected based on incidence of passively detected *Plasmodium falciparum* and mixed (*P. falciparum* and *P. vivax*) malaria cases during the 2013 malaria transmission season. All households in intervention *kebeles* were targeted; consenting residents received a rapid diagnostic test (RDT) and RDT-positive individuals received artemether-lumefantrine for *P. falciparum*/mixed infections or chloroquine for *P. vivax*. Data were collected on MTAT participation, sociodemographic characteristics, malaria risk factors, and RDT positivity.

**Results:**

Of 9162 households targeted, 7974 (87.0 %) participated in the MTAT. Among the 35,389 residents of these households, 30,712 (86.8 %) received an RDT. RDT-positivity was 1.4 % (0.3 % *P. vivax*, 0.7 % *P. falciparum*, 0.3 % mixed), ranging from 0.3 to 5.1 % by *kebele*; 39.4 % of RDT-positive individuals were febrile, 28.5 % resided in the same household with another RDT-positive individual, 23.0 % were not protected by vector control interventions [mosquito net or indoor residual spray (IRS)], and 7.1 % had travel history. For individuals under 10 years of age, the odds of being RDT-positive was significantly higher for those with fever, recent use of anti-malarial drugs or residing in the same household with another RDT-positive individual; 59.0 % of RDT-positive individuals had at least one of these risk factors. For individuals 10 years of age and older, the odds of being RDT positive was significantly higher for those with reported travel, fever, recent use of anti-malarial drugs, no use of vector control, and those residing in the same household as another RDT-positive individual; 71.2 % of RDT-positive individuals had at least one of these risk factors.

**Conclusions:**

In the Ethiopia setting, an MTAT intervention is operationally feasible and can be conducted with high coverage. RDT-positivity is low and varies widely by *kebele*. While several risk factors are significantly associated with RDT-positivity, there are still many RDT-positive individuals who do not have any of these risk factors. Strategies that target populations for testing and treatment based on these risk factors alone are likely to leave many infections undetected.

**Electronic supplementary material:**

The online version of this article (doi:10.1186/s12936-016-1333-3) contains supplementary material, which is available to authorized users.

## Background

More than 63 million people live in areas at risk of malaria in Ethiopia and more than 3 million presumed and confirmed cases of malaria were reported in 2013 [1]. Amhara National Regional State is the second-most populous region in Ethiopia and accounts for 31 % of the national malaria burden [2]. Malaria parasite prevalence in this region was estimated at 2 % in 2011 [3] and malaria accounted for 22 % of outpatient visits, 24 % of hospital admissions, and 10 % of health facility deaths in 2012 [4]. While the proportion of outpatient visits, hospital admissions and health facility deaths attributed to malaria has fallen to 7, 2 and 1 %, respectively, since 2012, malaria remains an important source of morbidity and mortality in the region [5, 6].

The Ethiopian Federal Ministry of Health is striving to achieve substantial progress toward malaria elimination in low transmission areas by 2020 [7]. Current malaria control strategies in Ethiopia include long-lasting insecticide-treated bed nets (LLINs), indoor residual spraying (IRS), case management and epidemic detection and response where appropriate [8]. To move toward malaria elimination, new strategies will need to be employed to address the large proportion of infections that remain asymptomatic or minimally symptomatic and never present for testing and treatment [9, 10].

Population-wide or mass approaches that target infected individuals for treatment with anti-malarial drugs regardless of whether they are symptomatic or present-to-care are a means to clear malaria parasites and reduce malaria transmission in this context [11]. Mass testing and treatment (MTAT) is the testing of an entire population with a point-of-care test and administration of treatment to individuals with a positive test result. Currently, rapid diagnostic tests (RDTs) are the only programme-relevant option available. In Ethiopia, the current first-line anti-malarial drug for *Plasmodium falciparum* and mixed (*P. falciparum* and *P. vivax*) malaria is artemether-lumefantrine with a regimen of six doses over 3 days, with multiple pills in each dose depending on weight or age [8]. Adherence to a six-dose regimen of artemether-lumefantrine has been found to be low in some settings [12, 13]. Given this context, MTAT with artemether-lumefantrine and RDTs, with attention to treatment adherence, might be a reasonable approach for parasite clearance in Ethiopia.

Published literature has reported on MTAT approaches to malaria parasite clearance in Burkina Faso, Zambia and Zanzibar [14–16], but no literature has described the implementation of a mass approach to clear malaria parasites from populations in Ethiopia. An MTAT campaign to clear malaria parasites from the population in low transmission areas in Amhara Region was conducted to evaluate its feasibility, impact and costs to inform the Government of Ethiopia’s strategy for malaria elimination. This paper presents the results of the operational feasibility and describes the profile of the targeted population and the risk factors associated with RDT-positivity.

## Methods

### Study sites

Six *kebeles* (villages) were purposively selected from a sample of 209 *kebeles* in eight districts in Amhara Region, Ethiopia, that collectively span the diverse ecological-epidemiological context across the region. Each of these *kebeles* is served by a health post. Health posts are primarily located in rural areas, typically staffed by two health extension workers (HEWs) employed by the Federal Ministry of Health, and serve the surrounding *kebele* with an approximate average population of 5000 people. Low transmission of both *P. falciparum* and *P. vivax* occur in the area. At the health posts in these 209 *kebeles*, community-based surveillance assistants work closely with HEWs, using mobile phones to upload information about malaria morbidity and commodities to the web-based District Health Information Software (DHIS 2) health information management system on a weekly basis.

Information on population, altitude and the number of RDT-confirmed *P. falciparum* and mixed malaria cases passively detected during the 2013 malaria transmission season, as reported in DHIS 2, was used to stratify *kebeles*. Strata included: (1) no transmission, with no *P. falciparum* or mixed malaria cases per 1000 population per week, (2) very low transmission, with >0 and <0.3 cases per 1000 population per week, (3) low transmission and high altitude, with ≥0.3 and <1 case per 1000 population per week and an altitude ≥2000 m, (4) low transmission and low altitude, with ≥0.3 and <1 case per 1000 population per week and an altitude <2000 m and, (5) moderate transmission, with ≥1 case per 1000 population per week.

**Fig. 1:**
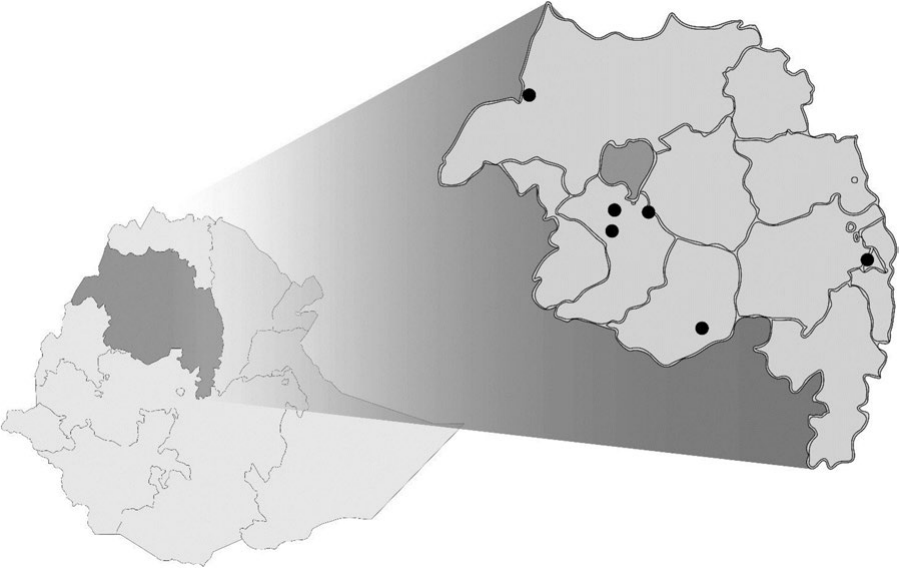
Map of study area. Showing the location of six health posts serving the *kebeles* that received a malaria MTAT intervention in Amhara Region, Ethiopia

Two *kebeles* were purposively selected from each of the three higher transmission strata (strata 3–5) based on accessibility, population mobility and the quality of the malaria morbidity data reported in DHIS 2. The entire *kebele* was targeted to receive the MTAT intervention. Sites included two *kebeles* in Bahir Dar Zuriya District (Dehina Sositu and Yeginid Lomi) and one *kebele* each in Mecha District (Berhan Chora), Aneded District (Zengoba), Kalu District (Choresa) and Metema District (Kumer Aftit) (Fig. 1). The population of each *kebele* ranged from 3441 to 8768 people; the combined population of all six *kebeles* was 38,530 in 2014 [17].

### Intervention design

One round of MTAT for malaria was conducted in the six *kebeles* at the beginning of the 2014 malaria transmission season, from late August through early September. Prior to the MTAT implementation, *kebele* community leaders and members of the Health Development Army conducted sensitization activities to inform the community about the objectives of the MTAT intervention. All households in the selected *kebeles* were censused (enumerated and geo-referenced). A total of 91 field teams of two surveillance assistants each went door to door to all households over a three-week period. The number of field teams in a given *kebele* ranged from 9 to 19, depending on the size and topography of the *kebele*. Field teams were supervised by staff from the district, zonal and regional health offices and the primary health care unit responsible for each health post.

All households in the selected *kebeles* were targeted with the aim to visit and test with RDTs the entire population older than 6 months of age and to provide anti-malarial treatment to all individuals testing positive. All households were approached by the field teams and invited to participate. The purpose of the intervention was explained and oral informed consent was obtained from all participants (or their parents or guardians if younger than 18 years old) prior to enrollment. At each household, the field team administered household and individual questionnaires and collected information on the geographic coordinates of the household (if not previously censused), the number of household members, mosquito net ownership, receipt of IRS in the previous 12 months, sociodemographic characteristics (age, sex, education, occupation) and individual risk factors for malaria (travel history, mosquito net usage, reported history of fever). Axillary temperature was measured using an electronic thermometer and recorded on the questionnaire. Reasons for non-participation were recorded for individuals who elected not to participate or who were not found at home. If any household members were absent at the time of the MTAT visit, the field team returned to the household up to two more times over the following 1 to 3 days to attempt to find the missing household members and conduct testing and treatment.

Available consenting household members older than 6 months of age were then tested with a CareStart™ Malaria Ag *P. falciparum*/*P. vivax* RDT for malaria. Individuals testing positive were treated with artemether-lumefantrine (*P. falciparum*, mixed) or chloroquine (*P. vivax*) according to national guidelines [8] and the RDT result and the anti-malarial treatment were also recorded in the questionnaire. Field teams provided instructions on how to take the anti-malarial treatment and observed the administration of the first dose of treatment.

Individuals with signs of serious illness, febrile individuals (axillary temperature ≥37.5 °C or reported fever in the last 24 h) who received anti-malarials in the previous 2 weeks and individuals with a positive RDT result who had treatment contra-indications were referred to the nearest health centre for evaluation and treatment according to national guidelines. Contra-indications to artemether-lumefantrine included first trimester of pregnancy, history of treatment with artemether-lumefantrine or oral quinine in the previous 2 weeks, or hypersensitivity to artemether and/or lumefantrine. Contra-indications to chloroquine included hypersensitivity to chloroquine, history of epilepsy and psoriasis.

Participants with *P. falciparum* and mixed malaria who received artemether-lumefantrine were followed up by field teams approximately 3 days after the initial MTAT visit to collect information in a standardized questionnaire on self-reported and observed adherence to treatment and the occurrence of adverse events. Any individuals with artemether-lumefantrine treatment remaining at the time of the follow-up visit were advised to complete treatment according to national guidelines.

### Data collection and analysis

Data were collected using smart phones equipped with questionnaires developed using Open Data Kit (ODK) and a custom android application used to navigate to households. Completed questionnaires were either uploaded directly from smart phones to an ODK Aggregate server and data repository in real time or manually downloaded from the smart phones after data collection was complete, depending on internet connectivity.

Data were analysed in Stata 13.1 (© StataCorp, College Station, TX, USA). Coverage of the MTAT intervention was estimated by calculating the proportion of all enumerated households reached in each *kebele* and the proportion of individuals tested in each household reached. Descriptive statistics were used to create a profile of sociodemographic characteristics, malaria risk factors, RDT-positivity, anti-malarial treatment administration, and adherence to treatment for individuals participating in the MTAT.

Risk factors for RDT-positivity were assessed using multivariate logistic regression. Two models were run separately for under 10 years of age and 10 years of age and older because the definition of the education and occupation variables differs in children and adults. In children it is the mother’s or head of household’s education and/or occupation (only the head of household’s occupation was available) rather than the individual’s education and occupation that are relevant, whereas in older children and adults it is pertinent to include the individual’s education and occupation. Effect modification between age group (under 10 years of age, 10 years of age and older) and the individuals’ education and occupation were evaluated and a significant interaction supported the a priori plan to run the two models separately. A univariate analysis was first run to estimate the odds ratio (OR) for the association between each variable and RDT-positivity. A multivariate model with RDT-positivity as the dependent variable was then run including all variables that had a significant OR in the univariate analysis, the *kebele* and all the a priori confounders/risk factors to produce adjusted ORs.

### Ethics statement

The study protocol was approved by the Amhara National Regional State Health Bureau Research Ethics Review Committee and received a non-research determination from PATH. Oral informed consent was obtained from all study participants or their parent or guardian if the participant was less than 18 years of age.

## Results

### Study population and intervention coverage

**Fig. 2:**
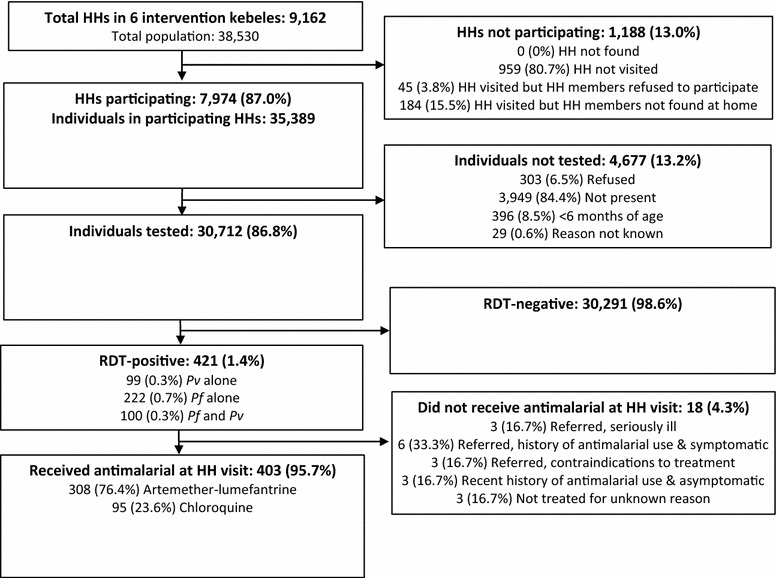
Study profile. Schematic of population reached by a malaria MTAT intervention in Amhara Region, Ethiopia. HH: household

Among 9162 enumerated households in the six *kebeles*, 7974 (87.0 %) participated in the MTAT intervention (Fig. 2). The majority (80.7 %) of non-participating households did not participate because they were never visited by a field team during the MTAT; the remaining households were visited by a field team but either refused to participate in the MTAT (3.8 %) or had no household members found at home (15.5 %).

There were 35,389 individuals residing in participating households, of which 30,712 (86.8 %) were tested for malaria. Among the 4677 individuals who were not tested during the MTAT, the majority (84.4 %) were not present in the household at the time of the MTAT; 6.5 % refused to participate, 8.5 % were younger than 6 months of age, and 0.6 % did not participate for an unknown reason.

The number of individuals tested in each *kebele* ranged from 3001 to 7765 (Table 1). Of the 30,712 individuals tested, 29.1 % were <10 years of age and 47.9 % were male; 78.1 % were protected by vector control interventions (slept under a mosquito net the previous night or resided in a household that received IRS in the previous 12 months), ranging from 17.8 to 92.3 % by *kebele*. Only 5.9 % of individuals were febrile, 1.4 % had a travel history (spent at least one night away from home in the previous month), 5.6 % resided in a household with at least one individual with a travel history, and 1.0 % resided in a household with more than one RDT-positive individual.

**Table 1:** Study population characteristics and RDT-positivity

	Total population tested with RDT		RDT-positive for *Pv* alone, *Pf* alone, or *Pf* and *Pv* (mixed)		
	N	Distribution of population tested (%)	n	% with positive RDT	Distribution of RDT-positive population (%)
Individuals tested with RDT	30,712	100.0	421	1.4	100.0
Geography					
Kebele 1 (Berhan Chora)	7188	23.4	21	0.3	5.0
Kebele 2 (Choresa)	3605	11.7	9	0.3	2.1
Kebele 3 (Dehina Sositu)	7765	25.3	61	0.8	14.5
Kebele 4 (Kumer Aftit)	3001	9.8	152	5.1	36.1
Kebele 5 (Yeginid Lomi)	5606	18.3	149	2.7	35.4
Kebele 6 (Zengoba)	3547	11.6	29	0.8	6.9
Sociodemographic characteristics					
Sex					
Female	15,991	52.1	181	1.1	43.0
Male	14,721	47.9	240	1.6	57.0
Age (years)					
<5	3694	12.0	58	1.6	13.8
5–9	5244	17.1	103	2.0	24.5
10–14	4224	13.8	58	1.4	13.8
15–19	3323	10.8	44	1.3	10.5
20–29	5045	16.4	91	1.8	21.6
30–39	3647	11.9	29	0.8	6.9
40–49	2404	7.8	22	0.9	5.2
50–59	1535	5.0	9	0.6	2.1
≥60	1596	5.2	7	0.4	1.7
Occupation					
No occupation	6525	21.3	104	1.6	24.7
Migrant labourer	164	0.5	8	4.9	1.9
Farmer	13,254	43.2	143	1.1	34.0
Student	6816	22.2	111	1.6	26.4
Housework	3072	10.0	39	1.3	9.3
Other	881	2.9	16	1.8	3.8
Education					
None	20,508	66.8	251	1.2	59.6
Primary school	8303	27.0	133	1.6	31.6
Secondary school or higher	1901	6.2	37	2.0	8.8
Malaria risk factors					
Vector control^a^					
No mosquito net or IRS	6703	21.8	97	1.5	23.0
Mosquito net and no IRS	8179	26.6	161	2.0	38.2
IRS and no mosquito net	3538	11.5	48	1.4	11.4
Mosquito net and IRS	12,292	40.0	115	0.9	27.3
Spent ≥1 night away from home in last month					
No	30,268	98.6	391	1.3	92.9
Yes	444	1.4	30	6.8	7.1
Febrile^b^					
No	28,888	94.1	255	0.9	60.6
Yes	1824	5.9	166	9.1	39.4
Took anti-malarial drugs in last 2 weeks					
No	30,672	99.9	410	1.3	97.4
Yes	40	0.1	11	27.5	2.6
>1 RDT-positive individual in household					
No	30,412	99.0	301	1.0	71.5
Yes	300	1.0	120	40.0	28.5
≥1 individual in household spent ≥ 1 night away from home in the last month					
No	28,986	94.4	378	1.3	89.8
Yes	1726	5.6	43	2.5	10.2

*Pf* *Plasmodium falciparum*; *Pv* *Plasmodium vivax*; *RDT* rapid diagnostic test

^a^Slept under a mosquito net last night and/or household received IRS in the last 12 months

^b^Measured fever (axillary temperature ≥37.5 °C) or history of fever in last 24 h

### Operational considerations

The 7974 participating households were visited by 91 field teams over 19 days. On average, each field team visited 8.4 households per day. The average household had 4.4 individuals. With 87.0 % of households in the intervention areas reached and 86.8 % of individuals in the households reached receiving an RDT, the effective coverage of the MTAT intervention was 75.5 %. Among the non-participating households, 80.7 % were never visited by the field workers, either because they were missed by the field teams, they were duplicated in the census, or they were visited but the field teams did not fill out a questionnaire with the reason for non-participation. The age and sex distribution of the individuals not found at home at the time of the MTAT was different than that of those found at home. The mean age of those absent was 26.4 years, compared to 22.8 years for those present and the percentage of males was 71.3 % and 48.0 % respectively, with 26.1 % of individuals not found at home being males between the ages of 15 and 29 years, compared to 11.9 % of individuals found at home.

### RDT-positivity

Of the 30,712 individuals with test results, 421 (1.4 %) were RDT-positive (0.3 % *P. vivax*, 0.7 % *P. falciparum* and 0.3 % mixed) (Fig. 2). Nearly all RDT-positive individuals (95.7 %) were treated with either artemether-lumefantrine or chloroquine.

### Factors associated with RDT-positivity

**Fig. 3:**
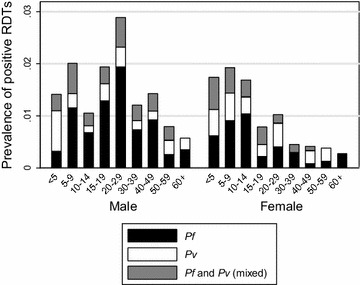
Prevalence of RDT-positivity. Prevalence of RDT-positivity for *P. falciparum*, *P. vivax* or *P. falciparum* and *P. vivax* (mixed) malaria by age and sex

RDT-positivity for any species (*P. falciparum*, *P. vivax* or mixed) varied by *kebele*, ranging from 0.3 to 5.1 %, and by other characteristics (Table 1). The RDT-positivity was 9.1 % in febrile individuals, 40.0 % in individuals who resided in the same household with another RDT-positive individual, 1.5 % in those not protected by vector control, 6.8 % in individuals who had travel history, 2.5 % in those who resided in the same household as at least one person with travel history and 27.5 % in those who had taken anti-malarial drugs in the previous 2 weeks. While RDT-positivity was low in all age groups, it was generally higher in males aged 5 to 29 years and females aged 6 months to 14 years (Fig. 3).

Factors associated with RDT-positivity for any species for individuals under 10 years of age and individuals 10 years of age and older are described in Tables 2 and 3, respectively. Factors associated with RDT-positivity by species (*P. falciparum*, *P. vivax* and mixed) are described in Additional file 1: Tables S1, S2. For individuals under 10 years of age, febrile individuals, individuals who took anti-malarial drugs in the last 2 weeks and individuals residing in a household with at least one other RDT-positive individual were significantly more likely to be RDT-positive for any species after adjusting for all other factors (Table 2); 59.0 % of RDT-positive individuals under 10 years of age had at least one of these risk factors.

**Table 2:** Odds of an RDT-positive result by risk factor for individuals under 10 years of age

	N (% of total population <10 years)	RDT-positive for *Pv* alone, *Pf* alone, or *Pf* and *Pv* (mixed)				
		n	% with positive RDT	Distribution of RDT-positive population (%)	Unadjusted OR (95 % CI)	Adjusted OR (95 % CI)^c^
Individuals tested with RDT	8938 (100)	161	1.8	100	–	–
Geography						
Kebele 1 (Berhan Chora)	2073 (23.2)	2	0.1	1.2	Ref	Ref ***
Kebele 2 (Choresa)	855 (9.6)	3	0.4	1.9	3.65 (0.61–21.86)	1.83 (0.27–12.56)
Kebele 3 (Dehina Sositu)	2625 (29.4)	32	1.2	19.9	12.78 (3.06–53.38)	6.74 (1.55–29.22)
Kebele 4 (Kumer Aftit)	850 (9.5)	37	4.4	23	47.13 (11.33–195.98)	30.00 (6.88–130.81)
Kebele 5 (Yeginid Lomi)	1555 (17.4)	76	4.9	47.2	53.21 (13.05–216.99)	32.04 (7.06–145.43)
Kebele 6 (Zengoba)	980 (11.0)	11	1.1	6.8	11.75 (2.60–53.14)	8.15 (1.75–39.94)
Sociodemographic characteristics						
Sex						
Female	4433 (49.6)	82	1.9	50.9	Ref	Ref
Male	4505 (50.4)	79	1.8	49.1	0.95 (0.71–1.27)	0.96 (0.68–1.39)
Age (years)						
<5	3694 (41.3)	58	1.6	36	Ref	Ref
5–9	5244 (58.7)	103	2	64	1.26 (0.98–1.62)	1.34 (0.92–1.96)
Occupation of household head						
No occupation	214 (2.4)	6	2.8	3.7	Ref	Ref
Migrant labourer	52 (0.6)	1	1.9	0.6	0.68 (0.07–6.97)	1.30 (0.11–15.3)
Farmer	6730 (75.3)	116	1.7	72.1	0.61 (0.21–1.74)	1.01 (0.35–2.86)
Student	45 (0.5)	1	2.2	0.6	0.79 (0.06–10.57)	1.92 (0.19–18.90)
Housework	212 (2.4)	5	2.4	3.1	0.84 (0.20–3.49)	1.27 (0.29–5.53)
Other or not known	1685 (18.9)	32	1.9	19.9	0.67 (0.28–1.61)	1.29 (0.43–3.82)
Malaria risk factors						
Vector control^1^						
No mosquito net or IRS	1829 (20.5)	35	1.9	21.7	Ref	Ref
Mosquito net and no IRS	2382 (26.7)	80	3.4	49.7	1.78 (0.60–5.26)	0.87 (0.50–1.50)
IRS and no mosquito net	1088 (12.2)	13	1.2	8.1	0.62 (0.13–2.91)	1.08 (0.50–2.31)
Mosquito net and IRS	3639 (40.7)	33	0.9	20.5	0.47 (0.11–1.96)	0.51 (0.28–0.94)
Spent ≥1 night away from home in last month						
No	8913 (99.7)	159	1.8	98.8	Ref	Ref
Yes	25 (0.3)	2	8	1.2	4.79 (0.71–32.48)	10.16 (1.18–87.58)
Febrile^2^						
No	8458 (94.6)	99	1.2	61.5	Ref	Ref***
Yes	480 (5.4)	62	12.9	38.5	12.52 (4.84–32.43)	15.08 (9.91–22.92)
Took antimalarial drugs in last 2 weeks						
No	8930 (99.9)	157	1.8	97.5	Ref	Ref*
Yes	8 (0.1)	4	50	2.5	55.88 (30.68–101.78)	12.47 (1.34–116.16)
>1 RDT-positive individual in household						
No	8834 (98.8)	106	1.2	65.8	Ref	Ref***
Yes	104 (1.2)	55	52.9	34.2	92.42 (27.09–315.34)	50.57 (30.23–84.58)
≥1 individual in household spent ≥ 1 night away from home in the last month						
No	8516 (95.3)	156	1.8	96.9	Ref	Ref
Yes	422 (4.7)	5	1.2	3.1	0.64 (0.39–1.05)	0.50 (0.15–1.72)

*CI* confidence interval; *IRS* indoor residual spraying; *OR* odds ratio; *Pf* *Plasmodium falciparum*; *Pv* *Plasmodium vivax*; *RDT* rapid diagnostic test; *Ref* reference

* p < 0.05, ** p < 0.01, *** p < 0.001 from likelihood ratio test

^a^Slept under a mosquito net last night and/or household received IRS in the last 12 months

^b^Measured fever (axillary temperature ≥ 37.5 °C) or history of fever in last 24 h

^c^Adjusted Odds Ratios for all sociodemographic characteristics and malaria risk factors

**Table 3:** Odds of an RDT-positive result by risk factor for individuals 10 years of age and older

	N (% of total population ≥ 10 years)	RDT-positive for *Pv* alone, *Pf* alone, or *Pf* and *Pv* (mixed)				
		n	% with positive RDT	Distribution of RDT-positive population (%)	Unadjusted OR (95 % CI)	Adjusted OR (95 % CI)^c^
Individuals tested with RDT	21,774 (100.0)	260	1.2	100.0	–	–
Geography						
Kebele 1 (Berhan Chora)	5115 (23.5)	19	0.4	7.3	Ref	Ref***
Kebele 2 (Choresa)	2750 (12.6)	6	0.2	2.3	0.59 (0.23–1.47)	0.57 (0.21–1.52)
Kebele 3 (Dehina Sositu)	5140 (23.6)	29	0.6	11.2	1.52 (0.85–2.72)	1.02 (0.54–1.93)
Kebele 4 (Kumer Aftit)	2151 (9.9)	115	5.4	44.2	15.15 (9.30–24.68)	13.33 (7.73–23.01)
Kebele 5 (Yeginid Lomi)	4051 (18.6)	73	1.8	28.1	4.92 (2.97–8.17)	3.04 (1.60–5.77)
Kebele 6 (Zengoba)	2567 (11.8)	18	0.7	6.9	1.89 (0.99–3.62)	1.42 (0.71–2.84)
Sociodemographic characteristics						
Sex						
Female	11,558 (53.1)	99	0.9	38.1	Ref	Ref*
Male	10,216 (46.9)	161	1.6	61.9	1.85 (1.41–2.44)	1.42 (1.04–1.93)
Age (years)						
10–14	4224 (19.4)	58	1.4	22.3	Ref	Ref***
15–19	3323 (15.3)	44	1.3	16.9	0.96 (0.70–1.33)	0.81 (0.51–1.28)
20–29	5045 (23.2)	91	1.8	35.0	1.32 (0.89–1.97)	1.10 (0.69–1.74)
30–39	3647 (16.8)	29	0.8	11.2	0.58 (0.42–0.80)	0.39 (0.22–0.72)
40–49	2404 (11.0)	22	0.9	8.5	0.66 (0.44–0.99)	0.52 (0.28–0.97)
50–59	1535 (7.1)	9	0.6	3.5	0.42 (0.20–0.90)	0.34 (0.15–0.77)
≥60	1596 (7.3)	7	0.4	2.7	0.32 (0.14–0.71)	0.32 (0.13–0.80)
Occupation						
No occupation	1063 (4.9)	14	1.3	5.4	Ref	Ref
Migrant labourer	156 (0.7)	8	5.1	3.1	4.05 (2.02–8.12)	2.46 (0.84–7.21)
Farmer	12,484 (57.3)	136	1.1	52.3	0.83 (0.47–1.44)	1.53 (0.81–2.90)
Student	5103 (23.4)	69	1.4	26.5	1.03 (0.56–1.90)	1.39 (0.67–2.87)
Housework	2661 (12.2)	30	1.1	11.5	0.85 (0.40–1.81)	1.01 (0.49–2.09)
Other	307 (1.4)	3	1.0	1.2	0.74 (0.11–4.76)	0.83 (0.21–3.21)
Education						
None	13,653 (62.7)	141	1.0	54.2	Ref	Ref
Primary school	6666 (30.6)	90	1.4	34.6	1.31 (0.91–1.90)	1.05 (0.71–1.57)
Secondary school or higher	1455 (6.7)	29	2.0	11.2	1.95 (1.07–3.54)	1.09 (0.65–1.84)
Malaria risk factors						
Vector control^a^						
No mosquito net or IRS	4874 (22.4)	62	1.3	23.9	Ref	Ref***
Mosquito net and no IRS	5797 (26.6)	81	1.4	31.2	1.10 (0.33–3.67)	0.81 (0.52–1.24)
IRS and no mosquito net	2450 (11.3)	35	1.4	13.5	1.12 (0.29–4.36)	0.70 (0.42–1.19)
Mosquito net and IRS	8653 (39.7)	82	1.0	31.5	0.74 (0.21–2.63)	0.40 (0.26–0.61)
Spent ≥1 night away from home in last month						
No	21,355 (98.1)	232	1.1	89.2	Ref	Ref***
Yes	419 (1.9)	28	6.7	10.8	6.52 (2.56–16.63)	6.07 (2.48–14.81)
Febrile^b^						
No	20,430 (93.8)	156	0.8	60.0	Ref	Ref***
Yes	1344 (6.2)	104	7.7	40.0	10.90 (4.60–25.85)	10.77 (7.93–14.62)
Took antimalarial drugs in last 2 weeks						
No	21,742 (99.9)	253	1.2	97.3	Ref	Ref***
Yes	32 (0.1)	7	21.9	2.7	23.78 (9.52–59.41)	9.83 (3.35–28.81)
>1 RDT-positive individual in household						
No	21,578 (99.1)	195	0.9	75.0	Ref	Ref***
Yes	196 (0.9)	65	33.2	25.0	54.41 (30.82–96.06)	26.57 (17.66–39.97)
≥1 individual in household spent ≥ 1 night away from home in the last month						
No	20,470 (94.0)	222	1.1	85.4	Ref	Ref
Yes	1304 (6.0)	38	2.9	14.6	2.74 (1.17–6.39)	0.94 (0.44–2.02)

*CI* confidence interval; *IRS* indoor residual spraying; *OR* odds ratio; *Pf* *Plasmodium falciparum*; *Pv* *Plasmodium vivax*; *RDT* rapid diagnostic test; *Ref* reference

* p < 0.05, ** p < 0.01, *** p < 0.001 from likelihood ratio test

^a^Slept under a mosquito net last night and/or household received IRS in the last 12 months

^b^Measured fever (axillary temperature ≥ 37.5 °C) or history of fever in last 24 h

^c^Adjusted Odds Ratios for all sociodemographic characteristics and malaria risk factors

For individuals 10 years of age or older, individuals who had a travel history, were febrile, took anti-malarial drugs in the previous 2 weeks, or resided in a household with at least one other RDT-positive individual were significantly more likely to be RDT-positive for any species when compared to individuals without these characteristics after adjusting for all other factors (Table 3). Sleeping under a mosquito net the previous night and residing in a household that received IRS in the last 12 months was protective against RDT-positivity for any species. Of all RDT-positive individuals 10 years of age or older, 71.2 % had at least one risk factor (did not sleep under a mosquito net the previous night and did not reside in a household that received IRS in the last 12 months, had a travel history, had a fever, took anti-malarial drugs in the last 2 weeks, or resided in a household with more than one RDT-positive individual).

### Adherence to treatment and adverse events

Of the 308 individuals receiving artemether-lumefantrine, 253 (82.1 %) received a follow-up visit. Individuals were followed up an average of 3.6 days (range 1 to 11 days) after being tested. At the time of the follow-up visit, 224 individuals (88.5 %) reported having taken at least the first treatment dose. Treatment completion could only be assessed for the 96 individuals who were followed up more than 3 days after administration of the first treatment dose (when all treatment doses should have already been taken), of which 41 (42.7 %) reported having completed all doses of the treatment and 21 (21.9 %) were observed to have more than one dose of treatment remaining in the blister pack at the time of the follow-up visit. No individuals who received a follow-up visit reported any adverse event after taking any dose of artemether-lumefantrine.

## Discussion

The Ethiopian Federal Ministry of Health is striving to achieve substantial progress toward malaria elimination by 2020 [7]. As new malaria parasite clearance strategies are being considered to supplement current vector control and case management strategies, information from the implementation of a mass parasite clearance approach can help guide decision-making. This MTAT provided the first in-country experience to inform the Ministry of Health’s decision-making about mass malaria parasite clearance strategies and the findings have several implications for malaria elimination planning and programme implementation in Ethiopia.

The results show that it is feasible to implement a mass malaria parasite clearance intervention in a large geographic area over a short period of time. However, it is also resource intensive and the two-person field teams required additional people and training above and beyond existing human resources within the health system. The coverage of households (percentage of total households in the population that participated) and the coverage of individuals within households (percentage of individuals in visited households that were included) was high (87 % for both). The main reason for not including 13 % of the households in the area was that households were not visited (81 %), with refusals (4 %) and absent households (15 %) representing a much lower percentage. In the households that were visited, the main reason for not including 13 % of the members was that they were absent (84 %), while the rest were refusals (7 %) and exclusions based on age (infants younger than 6 months, 9 %). This resulted in an effective coverage of 75.5 % of all individuals in the targeted geographic areas, which was lower than desired. Thus greater efforts should be made to improve both the households’ and individuals’ coverage, that is visit every single household in the targeted areas and, for households that are visited but have absent members, try to track those individuals when they come back. These coverage estimates are comparable to published estimates of coverage for MTAT interventions implemented elsewhere. During an MTAT campaign in Zanzibar with two rounds of testing and treatment, 64 % of the population was reached during one or both rounds [16]. During an MTAT campaign in Zambia with three rounds of testing and treatment, 88 % of the population was reached in one or more rounds [15]. It is likely that a higher proportion of the targeted population (both households and individuals) would have been reached if multiple MTAT rounds had been implemented. While little information (age and sex) was collected about the individuals that were not reached during the MTAT implementation, these individuals differed from individuals who were reached, with more adults and a higher proportion of males, who are at higher risk of malaria infection. This suggests that a mass malaria parasite clearance approach with a door-to-door campaign may systematically miss important segments of the population if no efforts are made to reach individuals who are not at home at the time of the campaign. Additional mechanisms would be needed to increase the coverage, either by increasing the number of visits conducted to households with absent individuals and/or through strategies that specifically target mobile populations upon their return.

Malaria infection prevalence as measured by RDTs was low overall (1.4 %) and varied widely by *kebele*, ranging from 0.3 to 5.1 %. However, malaria prevalence was probably underestimated due to an imperfect RDT sensitivity. While RDTs generally have a high sensitivity in clinical settings, their detection limit in the field is around 100 parasites per µl [18]. Evidence indicates that sub-microscopic infections may be common in low prevalence settings and the intervention may have missed a substantial proportion of individuals with malaria infection [19]. In a recent MTAT campaign in Zanzibar, RDT sensitivity was estimated to be only 5.6 % for infections with any parasite density and 47.6 % for infections with parasite densities greater than 100 parasites per µl [16]. Thus without more sensitive point-of-care tests for malaria, a substantial proportion of malaria infections may remain undetected and untreated when using an MTAT approach for mass malaria parasite clearance. Mass drug administration is an alternative for clearing infections that would not otherwise be detected.

Several risk factors were associated with significantly higher odds of being RDT-positive. However, only 59 % to 71 % (in under ten and over 10 years of age respectively) of RDT-positive individuals had at least one of those risk factors. While information on the presence of these risk factors could be used to target populations at highest risk of malaria infection, screening for these risk factors would leave many infections undetected. These results also confirm that, even in low transmission areas, more than half (60.6 %) of RDT-positive individuals were asymptomatic. Interventions that are based on fever screening will miss many infections in such a setting [20]. Further, these results indicate that population movement is a driver of malaria transmission for individuals 10 years of age or older. An important income source for many families in the area is the seasonal agricultural work that young males do in other areas of Amhara with higher malaria transmission. Most leave for the farming season and come back home between September and December [21]. Thus the higher RDT-positivity among young adult males is probably a reflection of the large percentage of individuals in this age group who are returning migrant workers. Previous studies in Ethiopia have also found travel history to be a risk factor for malaria [22, 23], suggesting the importance of strategies that target migrant workers and other mobile populations to prevent the importation of malaria into their home communities. Also, given that intra-household transmission is a driver for individuals of all ages, mosquito net coverage and usage and IRS coverage need to be improved.

Across all *kebeles*, the field teams tested 73 RDT-negative individuals for every RDT-positive individual identified. This ratio ranged from 20 to 401 depending on *kebele*, suggesting that the efficiency of a mass malaria parasite clearance approach varies widely by geography, even within areas with consistently low rates of malaria. Focal malaria parasite clearance approaches that target specific sub-sets of the population based on proximity to passively detected malaria cases may be an efficient alternative to mass malaria parasite clearance approaches in areas with less malaria transmission [11]. The frequency of passively detected malaria cases can be used to determine which areas to target with population-wide *versus* focal approaches. However, as the risk factor analysis showed, any focal strategy that selects the sub-population to test based on specific characteristics will leave infections undetected in the population.

Only 52.7 % of RDT-positive individuals were infected by *P. falciparum* alone; 47.3 % had *P. vivax* or mixed malaria. Mass and focal malaria parasite clearance approaches that target *P. falciparum* alone will leave many transmissible *P. vivax* infections untreated. For Ethiopia to move to malaria elimination, strategies for malaria parasite clearance will require drugs that effectively clear both *P. falciparum* and *P. vivax* infections, including clearance of the persistent liver stages in *P. vivax*-infected individuals.

With population-wide treatment approaches using a multi-day and multi-dose drug, adherence to the full course of treatment is important. Low self-reported and observed treatment completion rates were found among individuals who received the six-dose regimen of artemether-lumefantrine and who were followed up more than 3 days after administration of their first dose. This is consistent with findings from a recent prospective observational study in Ethiopia that found only 38.7 % of patients to be ‘probably adherent’ to artemether-lumefantrine following administration of the drug in a routine care setting [12]. Additionally, field teams did not always directly observe the intake of the first dose as per protocol, with only 88.5 % of participants reporting to have taken the first dose. Poor adherence to artemether-lumefantrine will limit the impact of a mass malaria parasite clearance approach and attention to adherence is critical. New drugs are now available that require fewer doses (e.g., dihydroartemisinin-piperaquine) and these may provide important advantages for population-wide malaria clearance efforts.

The study had some limitations. First, the six intervention *kebeles* were purposively selected to represent variation in malaria transmission intensity, altitude, accessibility, and population mobility within Amhara Region. While results from a mass malaria parasite clearance approach in these *kebeles* provides insight into potential expansion into other parts of Ethiopia, these *kebeles* are not necessarily representative of Amhara Region or Ethiopia as a whole. Second, the effective coverage was lower than desired, with an estimated 24.5 % of the targeted population not reached, which indicates that greater efforts would be needed to target the hard-to-reach subgroups. Third, household enumeration was done for this study both prior to and during the intervention. The household enumeration was not validated using satellite images or other means and it is possible that some households were enumerated more than once and that some households were never enumerated. Thus the coverage estimates should be interpreted with caution.

## Conclusions

A population-wide malaria MTAT intervention is operationally feasible and can be conducted with high participation of those reached, although additional strategies to cover the hard-to-reach groups would be needed. RDT-positivity is low and varies widely by *kebele*. Population movement is a driver of malaria transmission for older individuals (10 years of age and older) while intra-household transmission is a driver for individuals of all ages. This suggests the importance of strategies that target migrant workers and other travelers as well as strategies to improve mosquito net usage and IRS coverage. While several factors are significantly associated with a higher odds of RDT-positivity, many RDT-positive individuals do not have these risk factors. Parasite clearance strategies that target populations for testing and treatment based on these risk factors alone are likely to leave many infections undetected.

## Additional files

10.1186/s13567-016-0342-0 Odds of a *Pv*, *Pf*, or mixed RDT-positive result by risk factor for individuals under 10 years of age. **Table S2.** Odds of a *Pv*, *Pf*, or mixed RDT-positive result by risk factor for individuals 10 years of age and older.

## Abbreviations

CI: confidence interval
DHIS 2: district health information software, version 2
HEW: health extension worker
IRS: indoor residual spraying
LLIN: long-lasting, insecticide-treated bed net
MTAT: mass testing and treatment
ODK: open data kit
OR: odds ratio
RDT: rapid diagnostic test
