# Supplementary material for: Mass testing and treatment for malaria in low transmission areas in Amhara Region, Ethiopia
Source: Malar J. 2016 Jun 2;15:305. doi: 10.1186/s12936-016-1333-3 (PMC4890322; doi:10.1186/s12936-016-1333-3)
Supplement: Supplementary file 1 — 10.1186/s13567-016-0342-0 Odds of a Pv, Pf, or mixed RDT-positive result by risk factor for individuals under 10 years of age. Table S2. Odds of a Pv, Pf, or mixed RDT-positive result by risk factor for individuals 10 years of age and older. [file 12936_2016_1333_MOESM1_ESM.docx]

**Mass testing and treatment for malaria in low transmission areas in Amhara Region, Ethiopia**

**Supplementary Appendix**

**Table S1. Odds of a *Pv*, *Pf*, or mixed RDT-positive result by risk factor for individuals under 10 years of age**

|  | | | **N** | **RDT-positive for *Pv* alone** | | | **RDT-positive for *Pf* alone** | | | **RDT-positive for *Pf* and *Pv* (mixed)** | | |
| --- | --- | --- | --- | --- | --- | --- | --- | --- | --- | --- | --- | --- |
|  |  |  |  | **n**  **(%)** | **Unadjusted**  **OR**  **(95% CI)** | **Adjusted**  **OR**  **(95% CI)^3^** | **n**  **(%)** | **Unadjusted**  **OR**  **(95% CI)** | **Adjusted**  **OR**  **(95% CI)^3^** | **n**  **(%)** | **Unadjusted**  **OR**  **(95% CI)** | **Adjusted**  **OR**  **(95% CI)^3^** |
| **Individuals tested with RDT** | | | 8,938 | 45 (0.5) | --- | --- | 71 (0.8) | --- | --- | 45 (0.5) | --- | --- |
| **Geography** | | |  |  |  |  |  |  |  |  |  |  |
|  | Kebele 1 (Berhan Chora) | | 2,073 | 1 (0.1) | Ref | Ref | 1 (0.1) | Ref | Ref*** | 0 (0.0) | Ref | Ref |
|  | Kebele 2 (Choresa) | | 855 | 0 (0.0) | --- | --- | 2 (0.2) | 4.86  (0.44-53.64) | 5.97  (0.45-79.79) | 1 (0.1) | 0.09  (0.01-0.67) | 0.10  (0.01-0.92) |
|  | Kebele 3 (Dehina Sositu) | | 2,625 | 12 (0.5) | 9.52  (1.24-73.24) | 3.36  (0.42-27.25) | 9 (0.3) | 7.13  (0.90-56.31) | 6.25  (0.76-51.25) | 11 (0.4) | 0.32  (0.15-0.68) | 0.38  (0.14-1.07) |
|  | Kebele 4 (Kumer Aftit) | | 850 | 4 (0.5) | 9.80  (1.09-87.78) | 5.83  (0.60-56.28) | 20 (2.4) | 49.93  (6.69-372.59) | 48.77  (6.30-377.66) | 13 (1.5) | 1.19  (0.59-2.41) | 1.46  (0.42-5.04) |
|  | Kebele 5 (Yeginid Lomi) | | 1,555 | 18 (1.2) | 24.27  (3.24-181.96) | 3.49  (0.42-29.18) | 38 (2.4) | 51.90  (7.12-378.41) | 238.56  (24.65-2309.18) | 20 (1.3) | --- | --- |
|  | Kebele 6 (Zengoba) | | 980 | 10 (1.0) | 21.36  (2.73-167.10) | 10.82  (1.34-87.38) | 1 (0.1) | 2.12  (0.13-33.87) | 2.00  (0.12-32.33) | 0 (0.0) | --- | --- |
| **Sociodemographic characteristics** | | |  |  |  |  |  |  |  |  |  |  |
|  | Sex | |  |  |  |  |  |  |  |  |  |  |
|  |  | Female | 4,433 | 23 (0.5) | Ref | Ref | 35 (0.8) | Ref | Ref | 24 (0.5) | Ref | Ref |
|  |  | Male | 4,505 | 22 (0.5) | 0.94  (0.52-1.69) | 0.99  (0.53-1.83) | 36 (0.8) | 1.01  (0.63-1.61) | 0.97  (0.57-1.64) | 21 (0.5) | 0.86  (0.48-1.55) | 0.94  (0.48-1.83) |
|  | Age | |  |  |  |  |  |  |  |  |  |  |
|  |  | <5 years | 3,694 | 24 (0.7) | Ref | Ref | 17 (0.5) | Ref | Ref*** | 17 (0.5) | Ref | Ref |
|  |  | 5-9 years | 5,244 | 21 (0.4) | 0.61  (0.34-1.10) | 0.59  (0.32-1.09) | 54 (1.0) | 2.25  (1.30-3.89) | 2.59  (1.42-4.70) | 28 (0.5) | 1.16  (0.63-2.12) | 1.04  (0.53-2.04) |

**Table S1. Odds of a *Pv*, *Pf*, or mixed RDT-positive result by risk factor for individuals under 10 years of age (cont.)**

|  | | | **N** | **RDT-positive for *Pv* alone** | | | **RDT-positive for *Pf* alone** | | | **RDT-positive for *Pf* and *Pv* (mixed)** | | |
| --- | --- | --- | --- | --- | --- | --- | --- | --- | --- | --- | --- | --- |
|  |  |  |  | **n**  **(%)** | **Unadjusted**  **OR**  **(95% CI)** | **Adjusted**  **OR**  **(95% CI)^3^** | **n**  **(%)** | **Unadjusted**  **OR**  **(95% CI)** | **Adjusted**  **OR**  **(95% CI)^3^** | **n**  **(%)** | **Unadjusted**  **OR**  **(95% CI)** | **Adjusted**  **OR**  **(95% CI)^3^** |
|  | Occupation of household head | |  |  |  |  |  |  |  |  |  |  |
|  |  | No occupation | 214 | 1 (0.5) | Ref | Ref | 3 (1.4) | Ref | Ref | 2 (0.9) | Ref | Ref |
|  |  | Migrant laborer | 52 | 1 (1.9) | 4.18  (0.25-67.90) | 3.97  (0.17-94.05) | 0 (0.0) | --- | --- | 0 (0.0) | --- | --- |
|  |  | Farmer | 6,730 | 35 (0.5) | 1.11  (0.15-8.17) | 1.87  (0.22-15.24) | 55 (0.8) | 0.58  (0.18-1.87) | 0.74  (0.18-2.96) | 26 (0.4) | 0.41  (0.10-1.74) | 1.02  (0.16-6.41) |
|  |  | Student | 45 | 0 (0.0) | --- | --- | 1 (2.2) | 1.60  (0.16-15.73) | 4.41  (0.36-54.75) | 0 (0.0) | --- | --- |
|  |  | Housework | 212 | 0 (0.0) | --- | --- | 3 (1.4) | 1.01  (0.20-5.06) | 0.78  (0.12-5.13) | 2 (0.9) | 1.01  (0.14-7.23) | 2.42  (0.21-27.58) |
|  |  | Other or not known | 1,685 | 8 (0.5) | 1.02  (0.12-8.16) | 1.88  (0.21-16.53) | 9 (0.5) | 0.38  (0.10-1.41) | 0.42  (0.09-1.91) | 15 (0.9) | 0.95  (0.21-4.19) | 3.17  (0.50-20.24) |
| **Malaria risk factors** | | |  |  |  |  |  |  |  |  |  |  |
|  | Vector control^1^ | |  |  |  |  |  |  |  |  |  |  |
|  |  | No mosquito net or IRS | 1,829 | 8 (0.4) | Ref | Ref*** | 14 (0.8) | Ref | Ref*** | 13 (0.7) | Ref | Ref* |
|  |  | Mosquito net and no IRS | 2,382 | 30 (1.3) | 2.90  (1.33-6.34) | 2.22  (0.92-5.37) | 29 (1.2) | 1.60  (0.84-3.03) | 0.36  (0.16-0.82) | 21 (0.9) | 1.24  (0.62-2.49) | 0.70  (0.26-1.87) |
|  |  | IRS and no mosquito net | 1,088 | 1 (0.1) | 0.21  (0.03-167) | 0.20  (0.02-1.65) | 8 (0.7) | 0.96  (0.40-2.30) | 5.48  (1.63-18.47) | 4 (0.4) | 0.52  (0.17-1.58) | 1.08  (0.31-3.77) |
|  |  | Mosquito net and IRS | 3,639 | 6 (0.2) | 0.38  (0.13-1.09) | 0.32  (0.10-0.98) | 20 (0.6) | 0.72  (0.36-1.42) | 2.75  (0.89-8.46) | 7 (0.2) | 0.27  (0.11-0.68) | 0.23  (0.08-0.71) |
|  | Spent ≥1 night away from home in last month | |  |  |  |  |  |  |  |  |  |  |
|  |  | No | 8,913 | 44 (0.5) | Ref | Ref | 70 (0.8) | Ref | Ref | 45 (0.5) | Ref | Ref |
|  |  | Yes | 25 | 1 (4.0) | 8.40  (1.11-63.45) | 7.04  (0.32-153.34) | 1 (4.0) | 5.26  (0.70-39.44) | 4.73  (0.25-89.64) | 0 (0.0) | --- | --- |
|  | Febrile^2^ | |  |  |  |  |  |  |  |  |  |  |
|  |  | No | 8,458 | 31 (0.4) | Ref | Ref*** | 43 (0.5) | Ref | Ref*** | 25 (0.3) | Ref | Ref*** |
|  |  | Yes | 480 | 14 (2.9) | 8.17  (4.31-15.46) | 5.63  (2.76-11.50) | 28 (5.8) | 12.12  (7.46-19.69) | 10.37  (5.76-18.66) | 20 (4.2) | 14.67  (8.09-26.60) | 10.44  (5.07-21.48) |

**Table S1. Odds of a *Pv*, *Pf*, or mixed RDT-positive result by risk factor for individuals under 10 years of age (cont.)**

|  | | | **N** | **RDT-positive for *Pv* alone** | | | **RDT-positive for *Pf* alone** | | | **RDT-positive for *Pf* and *Pv* (mixed)** | | |
| --- | --- | --- | --- | --- | --- | --- | --- | --- | --- | --- | --- | --- |
|  |  |  |  | **n**  **(%)** | **Unadjusted**  **OR**  **(95% CI)** | **Adjusted**  **OR**  **(95% CI)^3^** | **n**  **(%)** | **Unadjusted**  **OR**  **(95% CI)** | **Adjusted**  **OR**  **(95% CI)^3^** | **n**  **(%)** | **Unadjusted**  **OR**  **(95% CI)** | **Adjusted**  **OR**  **(95% CI)^3^** |
|  | Took antimalarial drugs in last 2 weeks | |  |  |  |  |  |  |  |  |  |  |
|  |  | No | 8,930 | 44 (0.5) | Ref | Ref | 69 (0.8) | Ref | Ref* | 44 (0.5) | Ref | Ref |
|  |  | Yes | 8 | 1 (12.5) | 28.85  (3.47-239.42) | 2.08  (0.17-25.47) | 2 (25.0) | 42.81  (8.49-215.82) | 13.01  (1.81-93.67) | 1 (12.5) | 28.85  (3.48-239.43) | 0.42  (0.02-7.59) |
|  | >1 RDT-positive individual in household | |  |  |  |  |  |  |  |  |  |  |
|  |  | No | 8,834 | 34 (0.4) | Ref | Ref*** | 48 (0.5) | Ref | Ref*** | 24 (0.3) | Ref | Ref*** |
|  |  | Yes | 104 | 11 (10.6) | 30.61  (15.05-62.26) | 13.27  (5.64-31.23) | 23 (22.1) | 51.97  (30.20-89.46) | 17.72  (9.18-34.22) | 21 (20.2) | 92.88  (49.75-173.39) | 33.25  (15.61-70.84) |
|  | ≥1 individual in household spent ≥1 night away from home in the last month | |  |  |  |  |  |  |  |  |  |  |
|  |  | No | 8,516 | 43 (0.5) | Ref | Ref | 68 (0.8) | Ref | Ref | 45 (0.5) | Ref | Ref |
|  |  | Yes | 422 | 2 (0.5) | 0.94  (0.22-3.89) | 0.57  (0.08-4.27) | 3 (0.7) | 0.89  (0.28-2.84) | 0.84  (0.19-3.64) | 0 (0.0) | --- | --- |
| CI: confidence interval; IRS: indoor residual spraying; OR: odds ratio;  *Pf*: *Plasmodium falciparum*; *Pv*: *Plasmodium vivax*; RDT: rapid diagnostic test; Ref: reference  *p<0.05, **p<0.01, ***p<0.001from likelihood ratio test  ^1^ Slept under a bednet last night and/or household received IRS in the last 12 months.  ^2^ Measured fever (axillary temperature ≥37.5 °C) or history of fever in last 24 hours.  ^3^ Adjusted for all sociodemographic characteristics and malaria risk factors. | | | | | | | | | | | | |

**Table S2. Odds of a *Pv*, *Pf*, or mixed RDT-positive result by risk factor for individuals 10 years of age and older**

|  | | | **N** | **RDT-positive for *Pv* alone** | | | **RDT-positive for *Pf* alone** | | | **RDT-positive for *Pf* and *Pv* (mixed)** | | |
| --- | --- | --- | --- | --- | --- | --- | --- | --- | --- | --- | --- | --- |
|  |  |  |  | **n**  **(%)** | **Unadjusted**  **OR**  **(95% CI)** | **Adjusted**  **OR**  **(95% CI)^3^** | **n**  **(%)** | **Unadjusted**  **OR**  **(95% CI)** | **Adjusted**  **OR**  **(95% CI)^3^** | **n**  **(%)** | **Unadjusted**  **OR**  **(95% CI)** | **Adjusted**  **OR**  **(95% CI)^3^** |
| **Individuals tested with RDT** | | | 21,774 | 54 (0.3) | --- | --- | 151 (0.7) | --- | --- | 55 (0.3) | --- | --- |
| **Geography** | | |  |  |  |  |  |  |  |  |  |  |
|  | Kebele 1 (Berhan Chora) | | 5,115 | 4 (0.1) | Ref | Ref*** | 10 (0.2) | Ref | Ref*** | 5 (0.1) | Ref | Ref |
|  | Kebele 2 (Choresa) | | 2,750 | 2 (0.1) | 0.93  (0.17-5.08) | 0.39  (0.07-2.29) | 4 (0.2) | 0.74  (0.23-2.37) | 1.01  (0.29-3.53) | 0 (0.0) | --- | --- |
|  | Kebele 3 (Dehina Sositu) | | 5,140 | 5 (0.1) | 1.24  (0.33-4.64) | 0.65  (0.16-2.62) | 14 (0.3) | 1.39  (0.62-3.14) | 1.07  (0.45-2.58) | 10 (0.2) | 1.99  (0.68-5.83) | 1.58  (0.49-5.00) |
|  | Kebele 4 (Kumer Aftit) | | 2,151 | 11 (0.5) | 6.57  (2.09-20.65) | 2.96  (0.86-10.17) | 76 (3.5) | 18.70  (9.65-36.22) | 17.79  (8.58-36.90) | 28 (1.3) | 13.48  (5.20-34.95) | 10.15  (3.61-28.56) |
|  | Kebele 5 (Yeginid Lomi) | | 4,051 | 16 (0.4) | 5.07  (1.69-15.17) | 1.61  (0.45-5.78) | 46 (1.1) | 5.86  (2.96-11.63) | 4.14  (1.72-9.95) | 11 (0.3) | 2.78  (0.97-8.02) | 1.72  (0.49-6.10) |
|  | Kebele 6 (Zengoba) | | 2,567 | 16 (0.6) | 8.01  (2.68-24.00) | 4.51  (1.41-14.37) | 1 (0.0) | 0.20  (0.03-1.55) | 0.16  (0.02-1.25) | 1 (0.0) | 0.40  (0.05-3.41) | 0.36  (0.04-3.18) |
| **Sociodemographic characteristics** | | |  |  |  |  |  |  |  |  |  |  |
|  | Sex | |  |  |  |  |  |  |  |  |  |  |
|  |  | Female | 11,558 | 29 (0.3) | Ref | Ref | 48 (0.4) | Ref | Ref** | 22 (0.2) | Ref | Ref |
|  |  | Male | 10,216 | 25 (0.2) | 0.98  (0.57-1.67) | 0.73  (0.40-1.34) | 103 (1.0) | 2.44  (1.73-3.44) | 1.72  (1.14-2.60) | 33 (0.3) | 1.70  (0.99-2.92) | 1.41  (0.74-2.68) |
|  | Age | |  |  |  |  |  |  |  |  |  |  |
|  |  | 10-14 years | 4,224 | 10 (0.2) | Ref | Ref | 36 (0.9) | Ref | Ref** | 12 (0.3) | Ref | Ref |
|  |  | 15-19 years | 3,323 | 9 (0.3) | 1.14  (0.46-2.82) | 1.17  (0.45-3.05) | 24 (0.7) | 0.85  (0.50-1.42) | 0.63  (0.34-1.15) | 11 (0.3) | 1.17  (0.51-2.65) | 1.05  (0.43-2.55) |
|  |  | 20-29 years | 5,045 | 21 (0.4) | 1.76  (0.83-3.74) | 1.85  (0.70-4.86) | 53 (1.1) | 1.24  (0.81-1.89) | 0.84  (0.47-1.52) | 17 (0.3) | 1.19  (0.56-2.49) | 0.99  (0.40-2.44) |
|  |  | 30-39 years | 3,647 | 3 (0.1) | 0.35  (0.10-1.26) | 0.39  (0.09-1.71) | 18 (0.5) | 0.58  (0.33-1.02) | 0.32  (0.15-0.68) | 8 (0.2) | 0.77  (0.32-1.89) | 0.67  (0.22-2.01) |
|  |  | 40-49 years | 2,404 | 5 (0.2) | 0.88  (0.30-2.57) | 1.19  (0.33-4.32) | 12 (0.5) | 0.58  (0.30-1.12) | 0.36  (0.16-0.82) | 5 (0.2) | 0.73  (0.26-2.08) | 0.74  (0.22-2.52) |
|  |  | 50-59 years | 1,535 | 4 (0.3) | 1.10  (0.12-3.52) | 1.30  (0.33-5.15) | 3 (0.2) | 0.23  (0.07-0.74) | 0.16  (0.05-0.58) | 2 (0.1) | 0.46  (0.10-2.04) | 0.52  (0.10-2.67) |

**Table S2. Odds of a *Pv*, *Pf*, or mixed RDT-positive result by risk factor for individuals 10 years of age and older (cont.)**

|  | | | **N** | **RDT-positive for *Pv* alone** | | | **RDT-positive for *Pf* alone** | | | **RDT-positive for *Pf* and *Pv* (mixed)** | | |
| --- | --- | --- | --- | --- | --- | --- | --- | --- | --- | --- | --- | --- |
|  |  |  |  | **n**  **(%)** | **Unadjusted**  **OR**  **(95% CI)** | **Adjusted**  **OR**  **(95% CI)^3^** | **n**  **(%)** | **Unadjusted**  **OR**  **(95% CI)** | **Adjusted**  **OR**  **(95% CI)^3^** | **n**  **(%)** | **Unadjusted**  **OR**  **(95% CI)** | **Adjusted**  **OR**  **(95% CI)^3^** |
|  |  | ≥60 years | 1,596 | 2 (0.1) | 0.53  (0.12-2.42) | 0.89  (0.16-4.86) | 5 (0.3) | 0.37  (0.14-0.93) | 0.31  (0.10-0.89) | 0 (0.0) | --- | --- |
|  | Occupation | |  |  |  |  |  |  |  |  |  |  |
|  |  | No occupation | 1,063 | 1 (0.1) | Ref | Ref | 7 (0.7) | Ref | Ref | 6 (0.6) | Ref | Ref |
|  |  | Migrant laborer | 156 | 0 (0.0) | --- | --- | 6 (3.9) | 6.03  (2.00-18.20) | 3.55  (0.93-13.57) | 2 (1.3) | 2.29  (0.46-11.44) | 0.75  (0.12-4.75) |
|  |  | Farmer | 12,484 | 33 (0.3) | 2.81  (0.38-20.60) | 2.69  (0.25-20.91) | 81 (0.7) | 0.99  (0.45-2.14) | 2.15  (0.90-5.11) | 22 (0.2) | 0.31  (0.13-0.77) | 0.47  (0.17-1.29) |
|  |  | Student | 5,103 | 15 (0.3) | 3.13  (0.41-23.73) | 2.28  (0.27-19.44) | 40 (0.8) | 1.19  (0.53-2.67) | 1.70  (0.64-4.52) | 14 (0.3) | 0.48  (0.19-1.26) | 0.56  (0.17-1.84) |
|  |  | Housework | 2,661 | 5 (0.2) | 2.00  (0.23-17.13) | 1.50  (0.16-13.88) | 16 (0.6) | 0.91  (0.37-2.22) | 1.30  (0.49-3.46) | 9 (0.3) | 0.60  (0.21-1.68) | 0.70  (0.23-2.18) |
|  |  | Other | 307 | 0 (0.0) | --- | --- | 1 (0.3) | 0.49  (0.06-4.02) | 0.66  (0.07-5.83) | 2 (0.7) | 1.16  (0.23-5.75) | 1.26  (0.23-6.87) |
|  | Education | |  |  |  |  |  |  |  |  |  |  |
|  |  | None | 13,653 | 26 (0.2) | Ref | Ref* | 85 (0.6) | Ref | Ref | 30 (0.2) | Ref | Ref |
|  |  | Primary school | 6,666 | 19 (0.3) | 1.50  (0.83-2.71) | 1.73  (0.78-3.84) | 53 (0.8) | 1.28  (0.91-1.81) | 0.90  (0.54-1.51) | 18 (0.3) | 1.23  (0.68-2.21) | 1.13  (0.50-2.55) |
|  |  | Secondary school or higher | 1,455 | 9 (0.6) | 3.26  (1.53-6.97) | 3.52  (1.41-8.77) | 13 (0.9) | 1.44  (0.80-2.59) | 0.72  (0.35-1.49) | 7 (0.5) | 2.20  (0.96-5.01) | 1.04  (0.38-2.85) |
| **Malaria risk factors** | | |  |  |  |  |  |  |  |  |  |  |
|  | Vector control^1^ | |  |  |  |  |  |  |  |  |  |  |
|  |  | No mosquito net or IRS | 4,874 | 18 (0.4) | Ref | Ref** | 30 (0.6) | Ref | Ref | 14 (0.3) | Ref | Ref |
|  |  | Mosquito net and no IRS | 5,797 | 21 (0.4) | 0.98  (0.52-1.84) | 0.78  (0.37-1.65) | 46 (0.8) | 1.29  (0.81-2.05) | 0.95  (0.52-1.74) | 14 (0.2) | 0.84  (0.40-1.76) | 0.70  (0.30-1.65) |
|  |  | IRS and no mosquito net | 2,450 | 3 (0.1) | 0.33  (0.10-1.12) | 0.21  (0.06-0.77) | 23 (0.9) | 1.53  (0.87-2.64) | 0.98  (0.49-1.99) | 9 (0.4) | 1.28  (0.55-2.96) | 0.67  (0.26-1.72) |
|  |  | Mosquito net and IRS | 8,653 | 12 (0.1) | 0.37  (0.18-0.78) | 0.25  (0.11-0.58) | 52 (0.6) | 0.98  (0.62-1.53) | 0.60  (0.34-1.08) | 18 (0.2) | 0.72  (0.36-1.46) | 0.34  (0.16-0.76) |

**Table S2. Odds of a *Pv*, *Pf*, or mixed RDT-positive result by risk factor for individuals 10 years of age and older (cont.)**

|  | | | **N** | **RDT-positive for *Pv* alone** | | | **RDT-positive for *Pf* alone** | | | **RDT-positive for *Pf* and *Pv* (mixed)** | | |
| --- | --- | --- | --- | --- | --- | --- | --- | --- | --- | --- | --- | --- |
|  |  |  |  | **n**  **(%)** | **Unadjusted**  **OR**  **(95% CI)** | **Adjusted**  **OR**  **(95% CI)^3^** | **n**  **(%)** | **Unadjusted**  **OR**  **(95% CI)** | **Adjusted**  **OR**  **(95% CI)^3^** | **n**  **(%)** | **Unadjusted**  **OR**  **(95% CI)** | **Adjusted**  **OR**  **(95% CI)^3^** |
|  | Spent ≥1 night away from home in last month | |  |  |  |  |  |  |  |  |  |  |
|  |  | No | 21,355 | 51 (0.2) | Ref | Ref | 133 (0.6) | Ref | Ref*** | 48 (0.2) | Ref | Ref* |
|  |  | Yes | 419 | 3 (0.7) | 3.01  (0.94-9.69) | 1.26  (0.22-7.20) | 18 (4.3) | 7.16  (4.33-11.83) | 7.64  (2.27-25.74) | 7 (1.7) | 7.54  (3.39-16.77) | 5.28  (0.96-28.83) |
|  | Febrile^2^ | |  |  |  |  |  |  |  |  |  |  |
|  |  | No | 20,430 | 37 (0.2) | Ref | Ref*** | 87 (0.4) | Ref | Ref*** | 32 (0.2) | Ref | Ref*** |
|  |  | Yes | 1,344 | 17 (1.3) | 7.06  (3.97-12.57) | 6.24  (3.31-11.74) | 64 (4.8) | 11.69  (8.42-16.22) | 9.90  (6.71-14.58) | 23 (1.7) | 11.10  (6.47-19.02) | 7.17  (3.95-13.03) |
|  | Took antimalarial drugs in last 2 weeks | |  |  |  |  |  |  |  |  |  |  |
|  |  | No | 21,742 | 54 (0.3) | Ref | Ref | 146 (0.7) | Ref | Ref*** | 53 (0.2) | Ref | Ref* |
|  |  | Yes | 32 | 0 (0.0) | --- | --- | 5 (15.6) | 27.39  (10.40-72.12) | 8.88  (2.52-31.23) | 2 (6.3) | 27.28  (6.36-117.07) | 8.37  (1.55-45.27) |
|  | >1 RDT-positive individual in household | |  |  |  |  |  |  |  |  |  |  |
|  |  | No | 21,578 | 40 (0.2) | Ref | Ref*** | 112 (0.5) | Ref | Ref*** | 43 (0.2) | Ref | Ref*** |
|  |  | Yes | 196 | 14 (7.1) | 41.42  (22.15-77.45) | 21.38  (10.16-44.98) | 39 (19.9) | 47.61  (32.02-70.80) | 17.09  (10.41-28.06) | 12 (6.1) | 32.66  (16.95-62.95) | 8.10  (3.73-17.57) |
|  | ≥1 individual in household spent ≥1 night away from home in the last month | |  |  |  |  |  |  |  |  |  |  |
|  |  | No | 20,470 | 48 (0.2) | Ref | Ref | 128 (0.6) | Ref | Ref | 46 (0.2) | Ref | Ref |
|  |  | Yes | 1,304 | 6 (0.5) | 1.97  (0.84-4.60) | 0.92  (0.27-3.16) | 23 (1.8) | 2.85  (1.82-4.46) | 0.80  (0.28-2.32) | 9 (0.7) | 3.09  (1.51-6.32) | 1.14  (0.26-5.06) |
| CI: confidence interval; IRS: indoor residual spraying; OR: odds ratio;  *Pf*: *Plasmodium falciparum*; *Pv*: *Plasmodium vivax*; RDT: rapid diagnostic test; Ref: reference  *p<0.05, **p<0.01, ***p<0.001 from likelihood ratio test  ^1^ Slept under a bednet last night and/or household received IRS in the last 12 months.  ^2^ Measured fever (axillary temperature ≥37.5 °C) or history of fever in last 24 hours.  ^3^ Adjusted for all sociodemographic characteristics and malaria risk factors. | | | | | | | | | | | | |
